# Convergence in Mobility Data Sets From Apple, Google, and Meta

**DOI:** 10.2196/44286

**Published:** 2023-06-22

**Authors:** Gustavo Sganzerla Martinez, David J Kelvin

**Affiliations:** 1 Department of Microbiology and Immunology Dalhousie University Halifax, NS Canada; 2 Department of Pediatrics Izaak Walton Killam Health Center Canadian Center for Vaccinology Halifax, NS Canada

**Keywords:** Google, Apple, Meta, COVID-19 mobility, COVID-19, mobility, data set, data, pattern, pandemic, mobile, operating system, system, validation, tool, asset

## Abstract

**Background:**

The higher movement of people was one of the variables that contributed to the spread of the infectious agent SARS-CoV-2 during the COVID-19 pandemic. Governments worldwide responded to the virus by implementing measures that would restrict people’s movements, and consequently, the spread of the disease. During the onset of the pandemic, the technology companies Apple, Google, and Meta used their infrastructure to anonymously gather mobility reports from their users.

**Objective:**

This study aims to compare mobility data reports collected by Apple, Google, and Meta (formerly Facebook) during the COVID-19 pandemic and a major winter storm in Texas in 2021. We aim to explore the hypothesis that different people exhibit similar mobility trends during dramatic events and to emphasize the importance of this type of data for public health measures. The study also aims to promote evidence for companies to continue releasing mobility trends data, given that all 3 companies have discontinued these services.

**Methods:**

In this study, we collected mobility data spanning from 2020 to 2022 from 3 major tech companies: Apple, Google, and Meta. Our analysis focused on 58 countries that are common to all 3 databases, enabling us to conduct a comprehensive global-scale analysis. By using the winter storm that occurred in Texas in 20201 as a benchmark, we were able to assess the robustness of the mobility data obtained from the 3 companies and ensure the integrity of our findings.

**Results:**

Our study revealed convergence in the mobility trends observed across different companies during the onset of significant disasters, such as the first year of the COVID-19 pandemic and the winter storm that impacted Texas in 2021. Specifically, we observed strong positive correlations (*r*=0.96) in the mobility data collected from different tech companies during the first year of the pandemic. Furthermore, our analysis of mobility data during the 2021 winter storm in Texas showed a similar convergence of trends. Additionally, we found that periods of stay-at-home orders were reflected in the data, with record-low mobility and record-high stay-at-home figures.

**Conclusions:**

Our findings provide valuable insights into the ways in which major disruptive events can impact patterns of human mobility; moreover, the convergence of data across distinct methodologies highlights the potential value of leveraging mobility data from multiple sources for informing public health decision-making. Therefore, we conclude that the use of mobility data is an asset for health authorities to consider during natural disasters, as we determined that the data sets from 3 companies yielded convergent mobility patterns. Comparatively, data obtained from a single source would be limited, and therefore, more difficult to interpret, requiring careful analysis.

## Introduction

The COVID-19 pandemic has affected people worldwide and presented major public health challenges for authorities. Early in 2020, shortly after the emergence of SARS-CoV-2, many countries imposed restrictive measures to limit the movement of populations, and consequently, limit the spread of SARS-CoV-2 infections and the COVID-19 disease [1]. To aid in evaluating the effect of restrictive measures, the tech industry rapidly responded by offering tools to quantify the movement of people using anonymized mobility data. In fact, higher movement patterns have been directly associated with COVID-19 transmission [2-4], rendering this measurement a powerful tool for public health authorities to understand the dynamics of populations when facing a disaster.

With the onset of COVID-19, tech companies, including Apple, Google, and Meta, provided data sets throughout the pandemic, with each company using different methodologies for obtaining mobility data. Such data can be used by scientists and health authorities to understand transmission patterns, identify hotspots, and potentially effectively control outbreaks. The importance of this data is seen as it provides a real-time snapshot of population dynamics. For instance, it was suggested that the early introduction of the B.1.1.529 (ie, Omicron) SARS-CoV-2 variant in Mexico was facilitated by higher movement observed in the end-of-year celebrations [5]. Moreover, in Canada, a so-called successful pandemic management was attributed to the fact that the movement patterns of people were highly reduced during the initial waves of SARS-CoV-2 [6]. Higher transmission of SARS-CoV-2 in India was attributed to an urban exodus of city dwellers that had to return to their villages after lockdown policies were implemented [7]. Apart from COVID-19, crisis management in other natural disasters can benefit from mobility data. For example, in the aftermath of the cyclone Gaja in south India (November 2018), mobility data were used to visualize where displaced people were relocating, so humanitarian efforts could be directed to those areas [8]. Importantly, the in- and outbound flow of people in particular areas during significant events can be quantified by services that capture mobility trends [9].

Comparing distinct data can provide a more comprehensive understanding of mobility trends, which can be crucial for public health measures. Data are commonly seen as abstractions of the real world. For instance, in applications that benefit from data, there is generally a need to promote validation with external data [10]; this reality is widely explored in clinical settings [11], genomic studies [12], among others. Consequently, a model that can react similarly upon different inputs strengthens the conclusions that are derived from such data.

This study aims to explore the hypothesis that different people exhibit similar mobility trends during dramatic events, such as the outbreak of an infectious disease or climatic disasters, and that these trends can be captured by companies such as Apple, Google, and Meta using distinct collection methodologies. To test this hypothesis, the study will compare mobility data reports collected by these companies during the first year of the COVID-19 pandemic and a major winter storm that hit Texas in 2021. Additionally, the study will observe mobility trends in countries with varying shares of the mobile operating system (MOS) penetration market. The ultimate goal of the study is to emphasize the importance of these types of data for public health measures and to provide evidence supporting the continued release of mobility trends data by companies, given that all 3 companies have discontinued these services.

## Methods

### Apple Mobility Trends Reports

Apple collected daily data from January 13, 2020, until April 14, 2022, in 63 countries or regions [13]. The company obtained the number of anonymous requests for directions (walking, driving, or using public transportation) made to the Apple Maps application, native to Apple’s MOS. The collected information was then compared against itself to reflect a change in the volume of people moving, in some cases including a minimum threshold for directions made per day. As of today, the data are not available any longer within the company website.

### Google Community Mobility Reports

Google made use of insights obtained from processing data from Google Maps, the company’s location service, to provide a response to health authorities regarding COVID-19 [14]. The data were collected from 15 February 15, 2020, to October 13, 2022. The mobility reports encompass data from 135 countries or regions with major metropolitan areas also included. A baseline was established using the data accumulated during the 5-week pre-pandemic period from January 3, 2020, to February 6, 2020. Whenever a user who chose to enable their location services visited a categorized location on Google Maps on a specific date, an anonymous record was assigned. The data were split into the following 6 categories: grocery and pharmacy, retail and recreation, parks, workplace, residential, and public transportation. Even though the company is no longer releasing updated data, their previously collected data set is publicly available within the company’s website.

### Movement Data From Meta

Non-public data were obtained from Meta [15] as part of an agreement with Dalhousie University. Meta collected data from 198 countries and territories from February 22, 2020, until May 22, 2022. Major regions targeted by Meta were divided into defined units (600 m^2^ 600 m^2^). Whenever a user had location services enabled and moved between 2 units, a movement record was assigned. Regions with 300 or less qualifying users in a 24-hour period were disregarded. A pre-pandemic baseline was established for each country; however, no details of the individual calculation of the baseline are present in Meta’s documentation.

### 2021 US Winter Storm

In 2021, a major winter storm struck North America. In the state of Texas, which hardly ever records snowfall, weather stations in the Dallas-Forth Worth airport recorded at least 139 hours of freezing or below freezing temperatures, characterizing an uncommon phenomena in the region [16]. During the storm, officially, a death toll of 151 was reported. The estimated cost of the storm in Texas was around US $295 billion dollars. For control purposes, we selected the state of Georgia as a representative state that recorded no snowfall in the period of February-March 2021. We inquired snowfall precipitation to data collected from the National Center for Environmental Information, which enables precipitation records of several weather stations in the Southern United States.

### MOS Penetration Data

We obtained data from the service StatCounter Global Stats [17] to select the market penetration of the MOS for Apple’s iOS and Google’s Android. The service is composed of bots installed in over 1.5 million websites. The bots gather stats on operating systems that are accessing a particular website. In this work, we selected the month of April 2022 to analyze the market share of different MOSs, which is when the first tech company (ie, Apple) stopped their mobility data collection. A total of 58 countries were analyzed, which were the countries present in all 3 mobility data sets. A breakdown of the countries is available in Multimedia Appendix 1.

### Data Processing and Statistical Analyses

Each one of the 3 companies provided their data in daily batches, which we treated in 7-day periods. As the data from the 3 companies followed different magnitudes, a scaling process was performed by using the scale function in R (version 4.1.2; R Foundation for Statistical Computing). The function calculates the mean and standard deviation of a vector then removes the mean from each element and divides it by the standard deviation. For comparison purposes, we selected the countries that were shared between the 3 data sets.

The statistical analyses performed in this study included Shapiro-Wilk test for data normality, Pearson correlation, as well as *t* test and Wilcoxon rank test for mean comparison. These analyses were performed using the R programming language (version 4.1.2; R Core Team) under the package rstatisx (version 0.7.0). All figures generated in this study were created using the R package ggplot2 (version 3.3.6).

### Ethical Considerations

The data collected from Apple, Google, and Meta users were already sampled, anonymized, and depersonalized. Data sources from Apple and Google are publicly obtainable; on the other hand, data from Meta are not publicly available. We, as research partners with Meta, have access only to data formatted to prevent reidentification, which we obtained through a partnership between Meta and author DJK (a licensee at Dalhousie University). Moreover, as users, we commit to using these data only for the purposes outlined in our agreement with Meta and we ensure that any conclusions drawn from these data are based on solely scientific practices.

## Results

### Mobility Data Well Represent the Initial Onset of the SARS-COV-2 Pandemic

**Figure 1:**
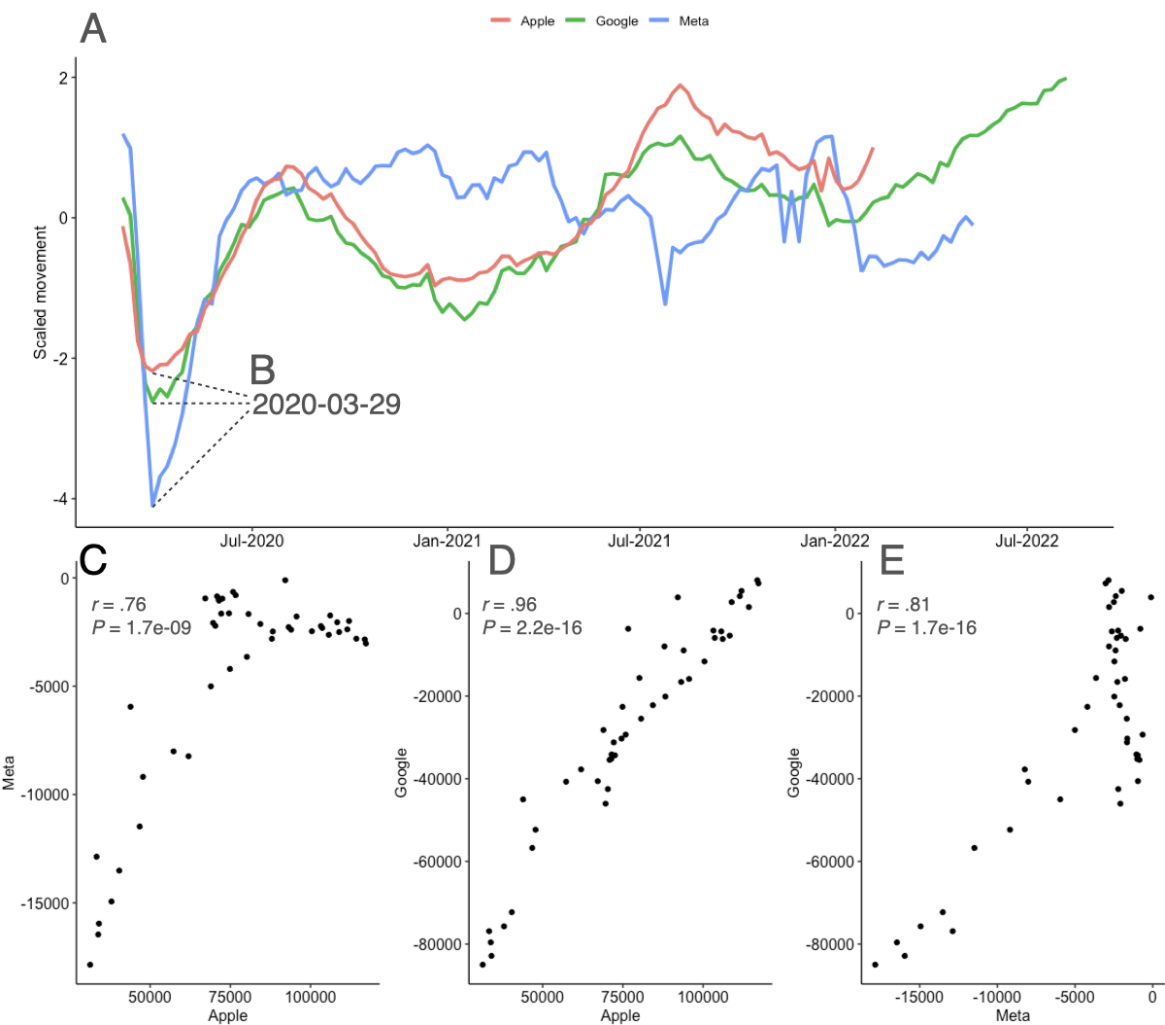
The 2020 mobility patterns from Apple, Google, and Meta across 58 countries. We selected the 58 countries that were present in the data from Apple, Google, and Meta. (A) From these countries, the year 2020 was isolated, and a mobility score for each company was assigned and later plotted. (B) The lowest mobility trend indicated in 2020 occurred in the same week for all 3 data sets, on March 29, 2020. (C) Next, we performed a Pearson correlation analysis of the data from Apple versus Meta, (D) Apple versus Google, and (E) Google versus Meta.

We used the data from the mobility reports of 58 countries collected by Apple, Google, and Meta to perform a comparative analysis to analyze people’s movements throughout the first year of the pandemic (ie, 2020; Figure 1). First, we noted the time series of each data collection methodology (ie, company) following the same trend (Figure 1A). Next, we analyzed the point at which each data set presented the lowest mobility score in the first year of the pandemic (Figure 1B). In 2020, the lowest mobility point for the 3 data sets (Figure 1A) occurred on March 29, 2020 (Figure 1B). Next, we performed a correlation analysis on the 3 data sets to depict their convergence or disparity (Figure 1C; Figure 1D, and Figure 1E); we report positive correlations for Apple versus Meta (*r*=0.76), Apple versus Google (*r*=0.95), and Google versus Meta (*r=*0.87), when comparing the data.

### The 2021 US Winter Storm Reflects in the Convergence of Mobility Data

**Figure 2:**
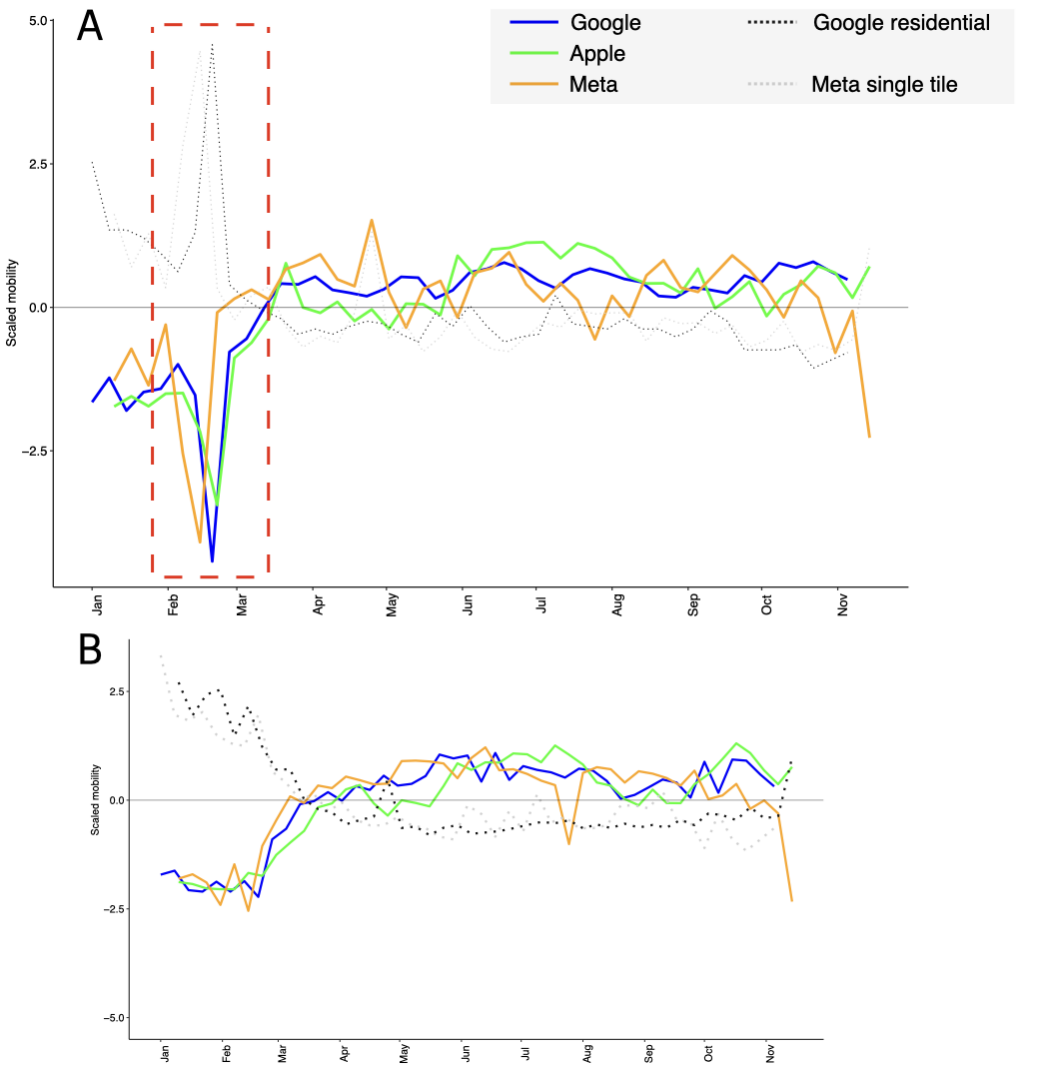
Record-low mobility trends recorded during a major winter storm in Texas in 2021. (A) Mobility data in the US state of Texas during the year of 2021. The negative area of the y-axis, indicating movement of data from 3 different tech companies—Apple (green solid line), Google (blue solid line), and Meta (orange solid line)—is considered. The data have been scaled to accommodate different magnitudes. The positive area of the y-axis indicates stay-at-home data, enabled only by Google (light dotted line) and Meta (dark dotted line). We also indicate the period in which a major winter storm in Texas took place (red dashed rectangle), from February 13 to 17, 2021. (B) A control version of part A, for the US state of Georgia. The state of Georgia presented no snow precipitation in the period from February 13 to 17, 2021.

We analyzed the movement data from Apple, Google, and Meta to determine the rate of individuals staying at home and not moving during the 2021 winter storm in the state of Texas. Figure 2A shows the period of the storm (depicted by the red-dashed rectangle), indicating low records in movement recorded by the 3 companies. We found the lowest mobility point occurred with no more than a week between each data set, specifically on February 21, 2021 (Apple), February 19, 2021 (Google), and February 14, 2021 (Meta). Additionally, we determined stay-at-home data from Google residential and Meta single tile users (represented by the grey dotted lines in Figure 2); these data were not available for Apple. Both companies noted record highs for stay-at-home data during the same days when record-low mobility was recorded. For control purposes, we selected the dates with the lowest mobility from each data set and compared them with data from the state of Georgia, which presented no snowfall precipitation during the 3 aforementioned days (Multimedia Appendix 2). In Figure 2B, we show that Georgia, unlike Texas, did not record a downfall in data on the dates of February 14, 19, and 21. Moreover, in Table 1, through a 2-sampled statistical test comparing the averages of mobility data from Apple, Google, and Meta over Texas and Georgia, we found low *P* values (Apple and Google: *P*<.001; Google and Meta: *P*=.005).

In Table 1, we present a comparison of the average movement during the months of January and February 2021 in the states of Texas and Georgia. The time series were found to be predominantly normally distributed through Shapiro-Wilk tests (Texas: *P*=.71 for Apple; *P*=.38 for Google; and *P*=.78 for Meta; Georgia: *P*=.71 for Apple; *P*=.04 for Google; and *P*=.51 for Meta). Moreover, the 2-sampled statistical comparison of the averages achieved by a 1-tailed *t* test resulted in statistically significant differences among all comparisons.

**Table 1:** Mean comparison of mobility trends during the Texas 2021 winter storm.

Data sets for 2 US states		Mobility average (January to February 2021)	*P* value	
**Apple**				<.001
	Texas	215147		
	Georgia	135228		
**Google**				<.001
	Texas	–497.4		
	Georgia	–250.8		
**Meta**				.005
	Texas	–3.15		
	Georgia	–0.81		

### Countries With Varied Market Penetration of MOS Have Convergent Mobility Data

**Figure 3:**
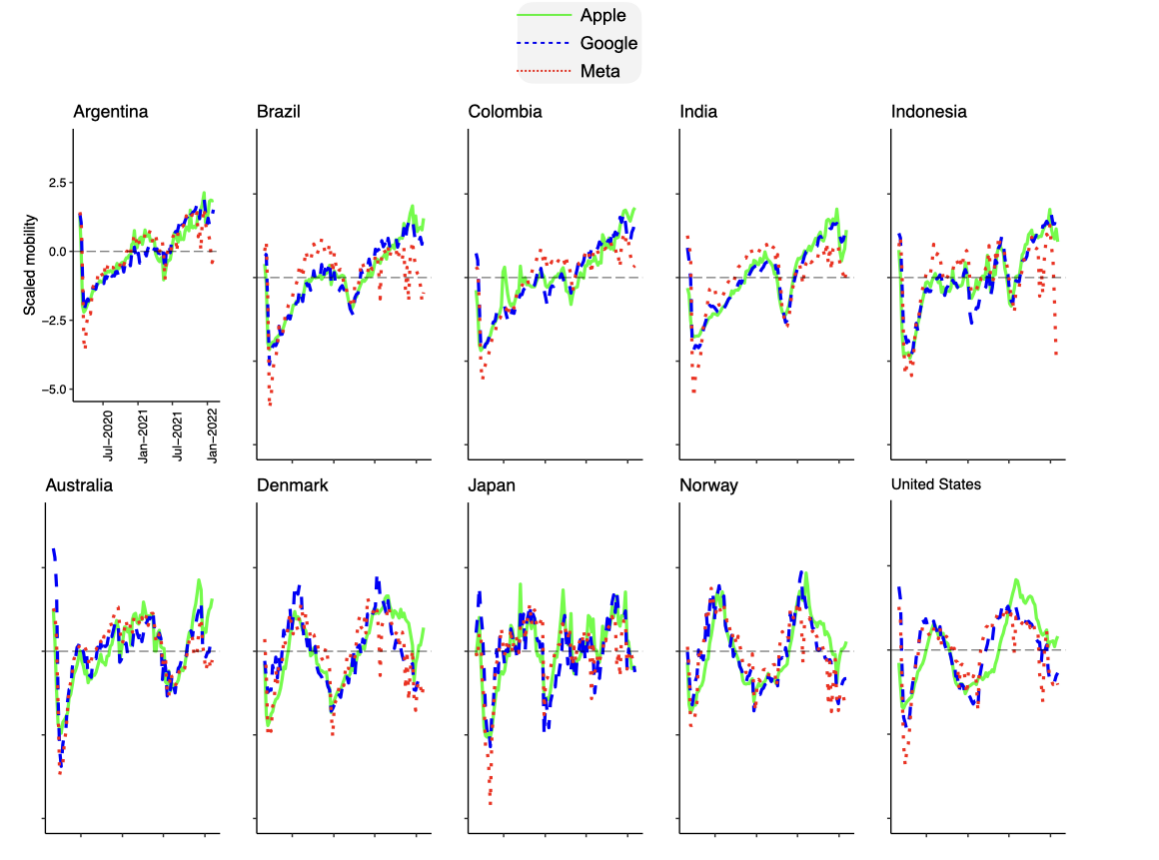
Comparison of mobility time series data from Apple, Google, and Meta in distinct countries. We selected the first 5 countries where Android is the most predominant mobile operating system (alphabetically, Argentina, Brazil, Colombia, India, and Indonesia) and the first 5 countries where iOS is the most predominant mobile operating system (alphabetically, Australia, Denmark, Japan, Norway, and the United States) according to data obtained from StatCounter Global Stats [17].

To determine if the predominance of an MOS would affect how mobility was recorded, we assessed the market share of mobile users regarding iOS (Apple) and Android (Google) from 54 countries that were common in the mobility data of Apple, Google, and Meta. We found that Android was the predominant platform in India, Argentina, Indonesia, Colombia, and Brazil, with greater than 90% of mobile internet access in April 2022, whereas iOS (Apple’s MOS) was the most popular platform in 5 countries—Japan, Denmark, Norway, United States, and Australia (67.6%, 60.3%, 59.3%, 57.8%, and 57.6%, respectively). To assess convergence or disparity between data collection methodologies, we individually correlated the 3 data sets in each country. In Figure 3, we present the time series data from the 5 iOS-predominant countries. The correlation table of these countries’ data is found in Table 2. The lowest averaged correlation was found in the United States (0.73, impacted by reduced correlation in the data of Apple and Meta), while the highest averaged correlation was found in Australia (80.66).

Also, in Figure 3, countries with Android predominance are shown and their correlation is presented in Table 2. First, we report an overall convergence in the mobility data collected by Apple and Google (ie, 0.85 average correlation across all the 10 countries considered). Second, we report a higher convergence between the data of Google and Meta (compared to Apple and Meta) in Android-dominated markets (except for Indonesia). This relationship may be attributed to the fact that Facebook is a third-party app that runs on an MOS, which in these countries is Android, thereby boosting the number of devices from which Google collects data. Finally, data from Apple was not as correlated with Meta as Google’s data in the iOS-prevalent countries, potentially due to the still higher proportion of Android devices being used in these countries.

**Table 2:** Correlation coefficients between data sets in different countries. To compare the time series between each data set, we performed Pearson correlation analyses for the iOS-prevalent countries and Android-prevalent countries.

Country	Comparison				Mobile operating system predominance
	Apple vs Google	Apple vs Meta	Google vs Meta		
Argentina	.91	.77	.84	Android	
Brazil	.96	.68	.75	Android	
Colombia	.91	.81	.86	Android	
India	.95	.77	.84	Android	
Indonesia	.93	.69	.64	Android	
Australia	.78	.76	.88	iOS	
Denmark	.76	.72	.79	iOS	
Japan	.79	.76	.75	iOS	
Norway	.84	.68	.87	iOS	
United States	.71	.59	.89	iOS	

## Discussion

### Principal Findings

In our work, we depicted a convergent behavior in mobility data gathered by distinct data collection methodologies independently of the popularity of MOSs. First, we showed that even over a longer 2-year time span, there is still a similar trend in mobility patterns, as evidenced by the moderate-to-strong correlation analyses in data collection from 3 distinct companies. Moreover, we observed that the mobility data converged more during events that required people to change their movement trends (ie, lockdowns, to avoid the spread of SARS-CoV-2; and a major winter storm that forced people to stay at home), granting this type of measure to be an asset for public health decisions.

We attribute the fact that we observed an alike behavior on data collected by different sources to 2 potential phenomena. First, when people are affected by a catastrophic event, such as the pandemic and the 2021 winter storm in Texas, their mobility and lifestyle will drastically change [10,18]. Considering a large number of people are experiencing the same stressor, the limited ways they develop to cope with stressful events, such as loneliness and social isolation, as reported by Hwang et al [19], will eventually converge. Moreover, it was reported in India and in the United Kingdom that a massive urban exodus took place during the pandemic, forcing many city-working young adults to have to return to their hometowns [20,21], despite being from different backgrounds, their mobility patterns might have converged once they were forced out of their workplaces. In this viewpoint, we argue that this convergence results from people developing ways to cope with their stress and will eventually be reflected in people moving in similar patterns. Next, users of distinct MOSs, whose data are being collected by distinct companies, might not significantly differ in terms of their movement patterns. In fact, behavioral similarities were previously reported among Android and iOS users, adding consistency to our analysis [22].

As a limitation of our study, we acknowledge that we did not have access to general mobility data from periods outside the pandemic, as these data were not being collected. Without these data, it was challenging to establish a solid baseline for comparison during and outside pandemic periods. It would have allowed us to gain a better understanding of the extent to which the observed behavior changes were driven by the pandemic itself or if they represented more general changes in human behavior over time. Furthermore, although smartphones have become increasingly prevalent in recent years, they do not represent the entire population. Therefore, we acknowledge that our sample may not have been a representative of the entire population. Specifically, individuals who do not own smartphones or have limited access to the internet might have different mobility patterns than those who do. Therefore, it is essential to consider the limitations of our sample when interpreting the results of our study. We believe these changes would lead to more robust policy and decision-making in public health.

The Data for Good portal [15], maintained by Meta with infrastructure from the company, provides a valuable data source for public health offices, as it collects data to support and inform efforts to tackle humanitarian and environmental challenges. As such, it is an asset for public health decision makers. For example, the company has been actively releasing their data at times of natural disasters to help inform decision makers, including during the flooding in Samar Island, Eastern Visayas, Philippines; the flooding in Southern California on January 5, 2023; the flooding in Makassar City, South Sulawesi, Indonesia; and the wildfire in Viña del Mar, Valparaíso, Chile, among others. As shown by our results, during these catastrophic events, the behavior of people might converge. Thus, the analysis of people’s movement might help answer important questions of public interest to reduce the impact of these events when they happen again.

### Conclusions

In our study, we observed that movement data collected under distinct methodologies by different tech companies presented a convergent aspect in depicting catastrophic events in which the movement of people was majorly impacted. The similar behaviors exhibited by different people facing similar stressors add reliability for these types of data, which would be an important asset upon which public health measures could rely. Thus, based on our results, we encourage Apple, Google, and Meta to continue releasing these types of data.

## Appendix

Multimedia Appendix 1 Intersection of countries that are shared between Apple, Google, and Meta mobility data sets.

Multimedia Appendix 2 The 2021 winter storm in southern United States.

## Abbreviations

MOS: mobile operating system

## Data Availability

The data sets generated and analyzed during this study are publicly available for Google [23] and Apple [24]. The Meta movement patterns were included in a private data set and obtained as part of a private agreement between Meta and Dalhousie University.
