# Supplementary material for: Convergence in Mobility Data Sets From Apple, Google, and Meta
Source: JMIR Public Health Surveill. 2023 Jun 22;9:e44286. doi: 10.2196/44286 (PMC10337444; doi:10.2196/44286)
Supplement: Multimedia Appendix 1 [file publichealth_v9i1e44286_app1.pdf]

innerj

| | country | continent | % apple | % android |
|---|---|---|---|---|
| 26 | Japan | AS | 67.6 | 32.28 |
| 12 | Denmark | EU | 60.33 | 39.25 |
| 2 | Australia | OC | 57.68 | 40.09 |
| 35 | Norway | EU | 59.35 | 40.16 |
| 56 | United States | NA | 57.87 | 41.83 |
| 8 | Canada | NA | 57.29 | 42.06 |
| 49 | Switzerland | EU | 56.4 | 42.86 |
| 48 | Sweden | EU | 54.96 | 44.53 |
| 55 | United Kingdom | EU | 53.58 | 45.91 |
| 19 | Hong Kong | AS | 49.59 | 48.5 |
| 41 | Saudi Arabia | AS | 49.42 | 50.45 |
| 23 | Ireland | EU | 48.43 | 51.14 |
| 34 | New Zealand | OC | 45.91 | 51.86 |
| 7 | Cambodia | AS | 42.2 | 57.37 |
| 4 | Belgium | EU | 39.22 | 59.96 |
| 33 | Netherlands | EU | 37.71 | 61.62 |
| 29 | Luxembourg | EU | 36.68 | 62.77 |
| 3 | Austria | EU | 36.13 | 62.95 |
| 15 | Finland | EU | 35.71 | 63.71 |
| 17 | Germany | EU | 32.44 | 64.84 |
| 14 | Estonia | EU | 34.44 | 65.05 |
| 32 | Morocco | AF | 32.9 | 66.62 |
| 16 | France | EU | 32.16 | 67.28 |
| 58 | Vietnam | AS | 31.93 | 67.53 |
| 51 | Thailand | AS | 29.02 | 70.63 |
| 40 | Russia | EU | 28.29 | 70.94 |
| 44 | Slovakia | EU | 28.29 | 70.94 |
| 27 | Latvia | EU | 27.47 | 71.81 |
| 25 | Italy | EU | 27.26 | 72.28 |

| | | | | |
|---|---|---|---|---|
| | South Korea | AS | 27.14 | 72.42 |
| 30 | Malaysia | AS | 25.28 | 73.34 |
| 43 | Singapore | AS | 25.27 | 73.82 |
| 54 | United Arab Emirates | AS | 25.64 | 74.09 |
| 24 | Israel | AS | 25.28 | 74.38 |
| 45 | Slovenia | EU | 24.99 | 74.61 |
| 20 | Hungary | EU | 22.27 | 77.06 |
| 28 | Lithuania | EU | 22.07 | 77.41 |
| 38 | Portugal | EU | 21.64 | 77.96 |
| 42 | Serbia | EU | 21.33 | 78.39 |
| 39 | Romania | EU | 21 | 78.68 |
| 47 | Spain | EU | 20.31 | 79.16 |
| 18 | Greece | EU | 17.37 | 80.82 |
| 53 | Ukraine | EU | 17.94 | 81.85 |
| 52 | Turkey | AS | 17.11 | 82.35 |
| 31 | Mexico | NA | 17.12 | 82.57 |
| 46 | South Africa | AF | 16.05 | 83.6 |
| 9 | Chile | SA | 15.21 | 84.39 |
| 57 | Uruguay | SA | 15.1 | 84.69 |
| 6 | Bulgaria | EU | 13.48 | 86.24 |
| 11 | Croatia | EU | 12.53 | 87.06 |
| 13 | Egypt | AF | 11.65 | 88.08 |
| 36 | Philippines | AS | 9.8 | 89.54 |
| 37 | Poland | EU | 10.21 | 89.6 |
| 5 | Brazil | SA | 9.38 | 90.48 |
| 10 | Colombia | SA | 8.87 | 90.78 |
| 22 | Indonesia | AS | 8.51 | 91.38 |
| 1 | Argentina | SA | 7.31 | 92.47 |
| 21 | India | AS | 3.83 | 94.91 |
