# Supplementary figures and images for: Convergence in Mobility Data Sets From Apple, Google, and Meta
Source: JMIR Public Health Surveill. 2023 Jun 22;9:e44286. doi: 10.2196/44286 (PMC10337444; doi:10.2196/44286)

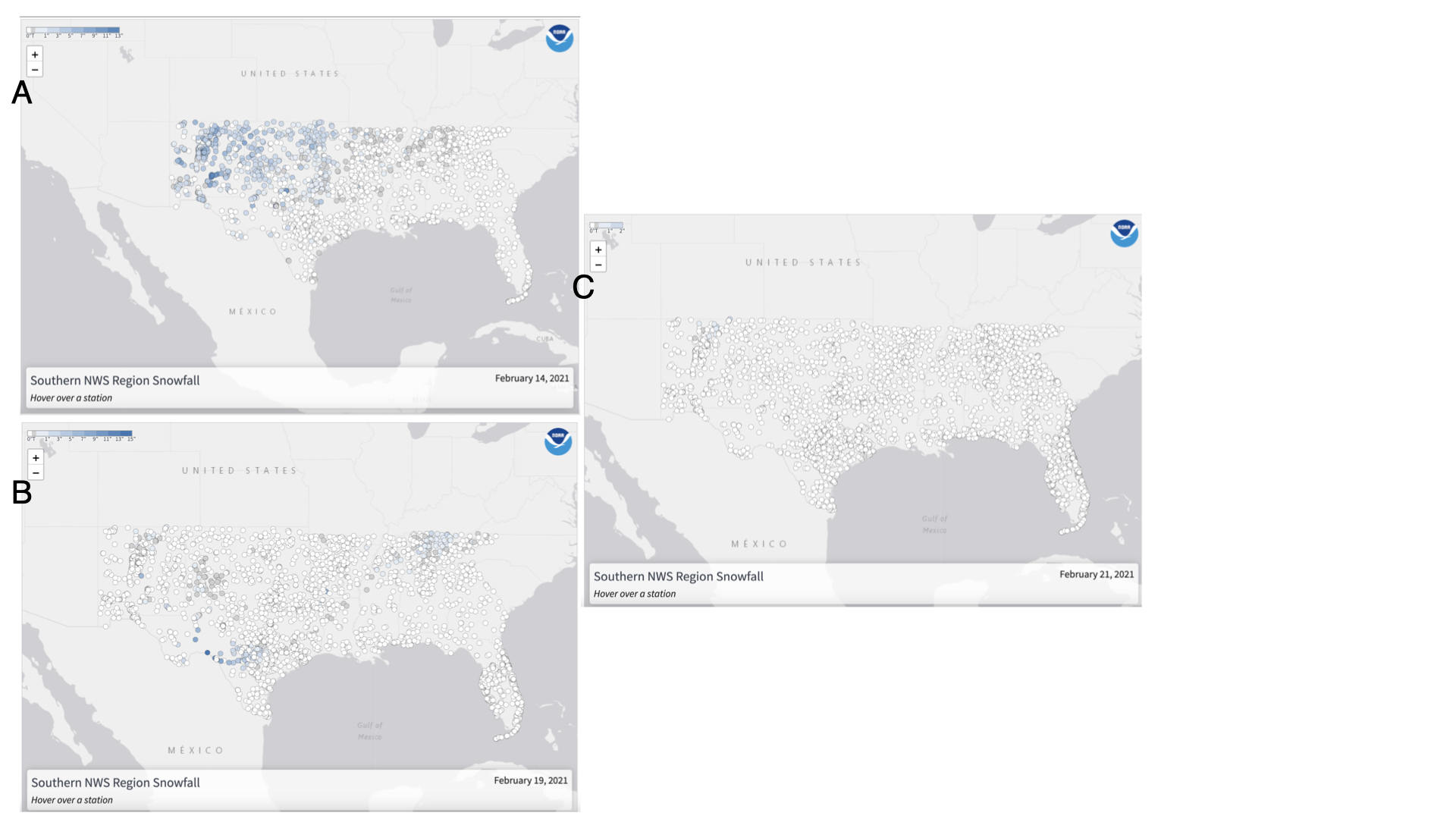

Supplement: Multimedia Appendix 2 [file publichealth_v9i1e44286_app2.png]
